# Non-invasive suppression of the human nucleus accumbens (NAc) with transcranial focused ultrasound (tFUS) modulates the reward network: a pilot study

**DOI:** 10.3389/fnhum.2024.1359396

**Published:** 2024-04-02

**Authors:** Xiaolong Peng, Dillon J. Connolly, Falon Sutton, John Robinson, Brenna Baker-Vogel, Edward B. Short, Bashar W. Badran

**Affiliations:** Department of Psychiatry and Behavioral Sciences, Neuro-X Lab, Medical University of South Carolina, Charleston, SC, United States

**Keywords:** focused ultrasound, tFUS, nucleus accumbens, reward network, fMRI

## Abstract

**Background:**

The nucleus accumbens (NAc) is a key node of the brain reward circuit driving reward-related behavior. Dysregulation of NAc has been demonstrated to contribute to pathological markers of addiction in substance use disorder (SUD) making it a potential therapeutic target for brain stimulation. Transcranial focused ultrasound (tFUS) is an emerging non-invasive brain stimulation approach that can modulate deep brain regions with a high spatial resolution. However, there is currently no evidence showing how the brain activity of NAc and brain functional connectivity within the reward network neuromodulated by tFUS on the NAc.

**Methods:**

In this pilot study, we carried out a single-blind, sham-controlled clinical trial using functional magnetic resonance imaging (fMRI) to investigate the underlying mechanism of tFUS neuromodulating the reward network through NAc in ten healthy adults. Specifically, the experiment consists of a 20-min concurrent tFUS/fMRI scan and two 24-min resting-state fMRI before and after the tFUS session.

**Results:**

Firstly, our results demonstrated the feasibility and safety of 20-min tFUS on NAc. Additionally, our findings demonstrated that bilateral NAc was inhibited during tFUS on the left NAc compared to sham. Lastly, increased functional connectivity between the NAc and medial prefrontal cortex (mPFC) was observed after tFUS on the left NAc, but no changes for the sham group.

**Conclusion:**

Delivering tFUS to the NAc can modulate brain activations and functional connectivity within the reward network. These preliminary findings suggest that tFUS could be potentially a promising neuromodulation tool for the direct and non-invasive management of the NAc and shed new light on the treatment for SUD and other brain diseases that involve reward processing.

## 1 Introduction

The reward circuit is formed by the interactions and integration of the mesolimbic, mesostriatal, and mesocortical pathways (Wise, 2009). The reward signal originates in the midbrain at the ventral tegmentum area (VTA) and partially in the substantia nigra (SN), and then mainly transmits via afferent synaptic projections to the nucleus accumbens (NAc), as well as the prefrontal cortex (PFC), amygdala, and hippocampus (Wise, 2009; Volkow et al., 2012; Cooper et al., 2017). The NAc is a key node of the brain reward circuit (Haber and Knutson, 2010; Koob and Volkow, 2010; Sesack and Grace, 2010; Cooper et al., 2017). The pattern of afferent and efferent synaptic connections elucidates the function of NAc as the site of information integration and interpretation, driving reward-related behavior, including encoding related sensory information into the context of reward, encoding receipt of reward, encoding expectancy of reward, guiding reward-driven behavior, determining behavioral response when presented with competing stimuli (Gardner, 2011; Floresco, 2015). Dysregulation of this region has been demonstrated to contribute to pathological markers of addiction such as cue reactivity and drug-seeking behavior in substance use disorder (SUD) making it a potential therapeutic target for the brain stimulation (Cooper et al., 2017).

Commonly used non-invasive brain stimulation (NIBS) techniques, such as transcranial direct current stimulation (tDCS) and transcranial magnetic stimulation (TMS) have shown to be efficacious as a treatment for select psychiatric disorders, particularly major depressive disorder (MDD) and obsessive-compulsive disorder (OCD) (George et al., 2009; Boes et al., 2018; Brunoni et al., 2019). For the treatment of SUD, these forms of NIBS have been used to target cortical regions, such as the dorsolateral prefrontal cortex (dlPFC), medial prefrontal cortex (mPFC), anterior cingulate cortex (ACC), and orbitofrontal cortex, that believe to have connections to the NAc, but only producing mostly suboptimal results or have a relatively short-lived effect of decreased craving levels for satiable foods and substances (Jansen et al., 2013; Grall-Bronnec and Sauvaget, 2014; Hone-Blanchet et al., 2015; Enokibara et al., 2016; Coles et al., 2018; Lapenta et al., 2018; Naish et al., 2018; Schluter et al., 2018; Luigjes et al., 2019). A possible explanation is that stimulation on these cortical regions can only deliver limited effects to the reward circuit-related subcortical regions. However, these forms of NIBS are limited in their application by depth and spatial resolution, making subcortical targets inaccessible (Polania et al., 2018).

Deep brain stimulation (DBS) of the NAc as a treatment for SUD is still in its preliminary stages, with only case reports and case series. Although early, the limited data available signals DBS of the NAc may be a procedure with significant therapeutic potential for the treatment of SUD, with long-term improvement or complete resolution in nearly all 25 subjects and remission rates seen as high as 50% at 6 years (Muller et al., 2013; Coles et al., 2018; Luigjes et al., 2019; Hassan et al., 2021). While promising, DBS requires an extensive invasive neurosurgical procedure with an implantable medical device, indicating long-term monitoring by a clinician. This exerts a substantial impact on the financial burden, safety profile, and overall feasibility (Coles et al., 2018; Luigjes et al., 2019).

A non-invasive form of DBS has emerged, known as transcranial focused ultrasound (tFUS), addresses the limitations of the above approaches and may prove to be an effective modality in the treatment of SUD. Previous clinical trials have demonstrated that tFUS can attenuate the sensory processing (Legon et al., 2014), pain (Badran et al., 2020), and modulate self-reported mood and mental vigor (Sanguinetti et al., 2020) by stimulating specific brain targets, including the thalamus and lateral frontal gyrus. Compared to other non-invasive stimulation approaches, tFUS has advantages in both stimulation depth and focal resolution. For example, tFUS has a deeper penetration range (stimulation depth: 1–12 cm) and a more focal spatial resolution (focality: <1 cm) than TMS (stimulation depth: 1–4 cm, focality: <3 cm) (Rezayat and Toostani, 2016; Badran and Peng, 2023). These advantages allow tFUS to modulate deep brain regions with a high spatial resolution which expands the scope of traditional non-invasive neuromodulation approaches to previously inaccessible regions, such as the NAc. Current non-invasive brain stimulation techniques cannot modulate the nucleus accumbens or other deep brain structures that are highly relevant in the treatment of substance use disorders, and thus investigating whether tFUS can produce biological effects directly on these deep brain structures provides a new opportunity to develop interventions that otherwise would be technologically limited. A recently published case report demonstrated that 10-min tFUS on NAc for a participant with SUD can greatly reduce (∼50%) the craving for the participant’s substances of use compared to sham, shedding new light on tFUS treatment for SUD (Mahoney et al., 2023). However, there is currently no evidence showing how the brain activity of NAc and brain functional connectivity within the reward network neuromodulated by tFUS on the NAc.

In this pilot study, we combine functional brain imaging techniques and tFUS to carry out a single-blind, sham-controlled clinical trial to investigate the underlying mechanism of tFUS neuromodulating the reward network through NAc in ten healthy adults. Specifically, we explored the direct tFUS-induced brain activity changes in NAc using a concurrent tFUS/fMRI system. Additionally, we also identified the functional connectivity changes between NAc and mPFC (two nodes of the reward network) using the resting-state fMRI data collected before and after tFUS on the NAc. These preliminary results from our present study will deepen our understanding of direct non-invasive neuromodulation for the reward circuits via NAc and serve as fundamental knowledge for effective brain intervention therapy for SUD.

## 2 Materials and methods

### 2.1 Participants

Ten healthy individuals (7 females, mean age ± SD: 31 ± 8.39 years) were recruited for this single-blind, sham-controlled, pilot study. Participants attended a single experimental visit during which they were randomly assigned to receive either active tFUS (*N* = 5, mean age ± SD: 31.2 ± 9.99 years) or sham tFUS (*N* = 5, mean age ± SD: 31.2 ± 7.66 years) with neuroimaging conducted before-, during- and after- tFUS administration. There is no difference in age between the two groups (two-sample *t*-test, *p* = 1). All participants were unaware of whether they were receiving active or sham tFUS.

Specifically, a structural T1-weighted magnetic resonance imaging (MRI) scan was first acquired, followed by pre-tFUS resting-state functional MRI (fMRI; four, 6-min scans). Next, 20 min of either active or sham tFUS was administered targeting the left NAc during concurrent fMRI acquisition (two, 10-min scans). Lastly, a post-tFUS resting-state fMRI was acquired (four, 6-min scans). This study was approved by the Institutional Review Board of the Medical University of South Carolina and registered on ClinicalTrials.gov (NCT05986019). All participants signed the informed consent before enrollment.

### 2.2 Concurrent tFUS/fMRI approach

#### 2.2.1 tFUS targeting in the MRI

**FIGURE 1:**
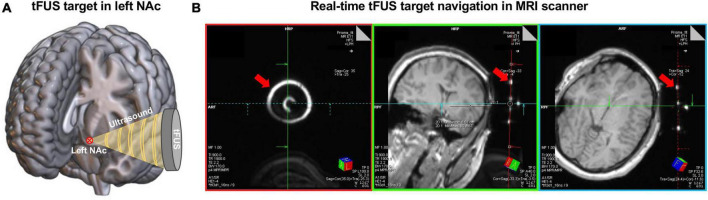
The procedure of tFUS target in left NAc. **(A)** Schematic of delivering ultrasound to the left NAc using tFUS. The red circled cross shows the location of the planned tFUS target—left NAc. **(B)** An example of the real-time tFUS target navigation procedure in the MRI scanner. These images were acquired from a rapid structure MRI sequence that captured the brain anatomy and the tFUS transducer. The bright circles and dots pointed by the red arrows show the location of the tFUS transducer. Digitized lines were created that intersect the fixed fiducials that are incorporated into the tFUS transducer. Specifically, the red line aligned with the back of the transducer. The blue line and the green line are orthogonal to the center of the transducer and extend into the left NAc target.

Real-time tFUS targeting was conducted within the bore of the MRI prior to ensuring the tFUS transducer was in the correct position to deliver ultrasound to the NAc target as described in our previous study (Badran et al., 2020). Specifically, the transducer was fixed on the participant’s scalp inside the scanner using a head-worn mount with Velcro straps that helped tune the location and angle of the transducer targeting the left NAc (Figure 1A). A rapid structural scout MRI sequence was scanned to capture both the brain anatomy and the tFUS transducer. Then, three colored digitized lines were created on the Siemens Prisma Scanner Console computer to help confirm the tFUS target location in the brain (Figure 1B). Each digitized line intersects and is orthogonal to each other. The green line and the red line were set intersected in the center of the tFUS transducer (Figure 1B, left), and the red line is aligned parallel to the surface of the transducer (Figure 1B, middle and right). Under this condition, the blue line in the middle panel of Figure 1B represents the direction of sonication propagation, and the location 65 mm underlying the skull on the blue line represents the stimulation focal point. The targeting was then confirmed by two experienced neuroimagers on the MRI Scanner Console computer in a real-time fashion to visually determine whether the 65 mm projected line intersects the planned target—left NAc. If the sonication trajectory was not confirmed on target, the position of the transducer would be manually adjusted to better align with the target until its structural target engagement is confirmed. The final mean distance (± SD) between the focal point of the tFUS beam and NAc target across 10 participants was 0.61 ± 1.17 mm.

#### 2.2.2 Concurrent tFUS/fMRI scan

After tFUS targeting, the concurrent tFUS-fMRI scan was performed. Each tFUS-fMRI run consisted of a 30s tFUS “ON” block, followed by a 30s “OFF” block, and repeated ten times. During the tFUS “ON” block, ultrasound stimulations were generated using the BrainSonix BXPulsar 1002 tFUS System (BrainSonix Corp., Sherman Oaks, CA, USA) with sonication parameters as follows: Fundamental frequency = 650 kHz, Pulse repetition frequency = 10 Hz, Pulse width = 5 ms, Duty cycle = 5%, Sonication duration = 30 s, ISPTA.0 = 995 mW/cm^2^, ISPTA.3 = 719 mW/cm^2^, Peak rarefactional pressure = 0.72 MPa. The transducer of this sonication system was a circular, single-element, air-backed, spherically focused transducer with a 61 mm active aperture and 65 mm focal length (as measured in water). The transducer was coupled to the scalp (unshaven) of the participant using a transducer holder that allows for sonication to occur in conjunction with ultrasound coupling gel. For the sham group, the tFUS system was set up identically to the active tFUS group, including targeting, however, tFUS was not turned on during the tFUS-fMRI scan. After the tFUS-fMRI session, the transducer was removed from the participant’s head, and the final session of resting-state fMRI was acquired.

### 2.3 MRI data acquisition

All MRI data were acquired on a Siemens 3T Prisma MRI scanner (Siemens; Erlangen, Germany). A 32-channel head coil was used for the T1-weighted structure MRI and the resting-state fMRI scans, while a 20-channel head coil was used for the concurrent tFUS/fMRI task scans to fit the tFUS transducer. The high-resolution structure images were collected using a T1-weighted MPRAGE sequence (TR = 2300 ms, TE = 2.26 ms, FA = 8°, 1.0 mm × 1.0 mm × 1.0-mm voxels, FOV 256). The structure images for tFUS targeting were acquired using a quick structural scout sequence (TR = 3150 ms, TE = 1.37 ms, FA = 8°, 1.6 mm × 1.6 mm × 1.6-mm voxels, FOV 128). Both resting-state fMRI and concurrent tFUS/fMRI were acquired using a gradient-echo echo-planar pulse sequence (TR = 2000 ms, TE = 36 ms, FA = 80°, 2.2 mm × 2.2 mm × 2.2-mm voxels).

### 2.4 MRI data pre-processing

#### 2.4.1 Structural MRI data pre-processing

Structural data were preprocessed using the FreeSurfer v5.3.0 software package.^1^ For each participant, the surface mesh of the cortical mantle was reconstructed from the structural T1-weighted image and then registered to a common spherical coordinate system (Dale et al., 1999).

#### 2.4.2 Resting-state fMRI data pre-processing

The resting-state fMRI data were preprocessed using a previously described analysis pipeline (Peng et al., 2023b), which included the following steps: (1) slice timing correction (Statistical Parametric Mapping, SPM2),^2^ (2) rigid body correction for head motion (FMRIB Software Library, FSL v5.0.4),^3^ (3) normalization for global mean signal intensity across runs, (4) bandpass filtering (0.01 to 0.08 Hz), and (5) nuisance signal regression of head-motion parameters and whole-brain, ventricular, and white matter signals. The preprocessed functional data were then registered to both the MNI152 template and the FreeSurfer “fsaverage6” cortical surface template, which consisted of 40,962 vertices in each hemisphere. Spatial smoothing was performed in surface space with a 6-mm full width at half maximum Gaussian kernel.

#### 2.4.3 Task fMRI data pre-processing

The MRI data were preprocessed using previously described procedures (Peng et al., 2023a), which included the following steps: (1) slice timing correction (Statistical Parametric Mapping, SPM2), (2) rigid body correction for head motion (FSL v5.0.4), (3) linear registration to the structural image (FreeSurfer), and (4) estimation of noise components using an iterative sparse noise modeling technique. Specifically, a “nuisance mask” that included white matter, cerebrospinal fluid, and non-brain tissues was defined according to gray matter probability and a grayscale threshold. Noise components were then learned from the signals extracted from the nuisance mask using an iterative sparse dictionary learning algorithm and specified as regressors of no interest in first-level modeling. Functional MRI responses to the tFUS task were analyzed by comparing “ON” stimulation blocks (when stimulation was being delivered during fixation) to “OFF” stimulation blocks which were fixation only using the following contrast “*tFUS ON* vs. *OFF*.” Contrasted task activations were estimated from a general linear model (GLM) by a restricted maximum likelihood approach (REML), in which the noise term was modeled using an AR (1) + white noise method, and then registered to the MNI152 template.

### 2.5 Statistical analysis

To investigate whether tFUS on left NAc directly induces brain activity changes in this area, first-level brain activation maps of “*tFUS ON–OFF*” were estimated for each single participant. A two-sample *t*-test (two-tailed) was then applied to calculate the second-level brain activation changes between tFUS active group and sham group within the NAc mask derived from the Harvard-Oxford cortical and subcortical structural atlases. Furthermore, to explore whether tFUS on NAc can modulate functional connectivity (FC) within the brain reward network, we, respectively, calculated the FC between the NAc and medial prefrontal cortex (mPFC) using the resting-state fMRI data acquired before and after the tFUS. A paired *t*-test (two-tailed) was then performed between pre- and post-tFUS in both active and sham groups to estimate the tFUS-induced FC changes.

## 3 Results

### 3.1 Safety of tFUS on the NAc

The tFUS parameters administered in this protocol, delivered for 20 min to the NAc were determined to be safe in this population. No adverse events were detected across all ten participants (5 tFUS active and 5 sham), and no reports of anhedonia or altered mental state were reported after completion of the tFUS.

### 3.2 tFUS of the left NAc inhibits bilateral NAc

**FIGURE 2:**
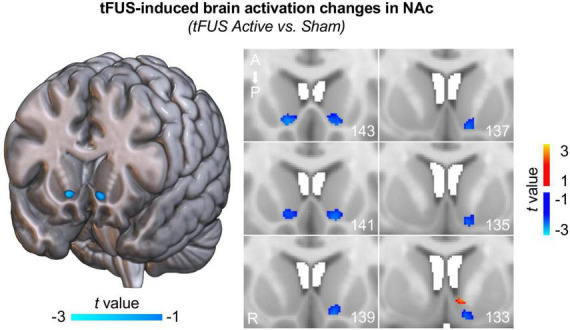
Transcranial focused ultrasound (tFUS) induces inhibited brain activation in NAc. Decreased brain activities were observed in the anterior part of the bilateral NAc and most regions of the left posterior NAc (two-sample *t*-test, *p* < 0.05, uncorrected) in the tFUS active group compared to the sham group, indicating that tFUS inhibited NAc activities. Note that, these deactivation maps were estimated based on group data comparison between active group and sham group within the NAc. Each figure on the right panel depicts different slices through the striatum. The numbers in the bottom right corners represent the slice number; ascending numbers go from anterior to posterior.

To investigate how brain activity changes in the NAc induced by direct tFUS on the left NAc, first-level brain activation maps of contrast “*tFUS ON* vs. *OFF*” were compared between the tFUS active group and the sham group within a bilateral NAc mask derived from the Harvard-Oxford subcortical atlas using a two-sample *t*-test. The second-level brain activation maps demonstrated that tFUS on the left NAc reduced the brain activities in the anterior part of the bilateral NAc and most regions of the left posterior NAc (Figure 2; two-sample *t*-test, *p* < 0.05), indicating that tFUS can directly inhibit NAc activities. It is worth noting that, these tFUS-induced brain activation changes in NAc cannot pass the correction for multiple comparisons due to the small sample size in this pilot study. To further investigate whether the tFUS target shift has potential effects on the region/nuclei near the target, we carried out a control analysis by comparing the mean brain activation changes within three adjacent striatal masks, including NAc, caudate, and putamen. We demonstrated that only mean brain activation within the NAc decreased in the active tFUS group compared to the sham, while increased brain activations of caudate and putamen were observed in the active tFUS group compared to the sham (Supplementary Figure 1). Considering we had a strong hypothesis for this pilot study that the tFUS will deactivate the NAc region due to the tFUS parameters we used (which have demonstrated suppressive effects in prior work), all the above evidence indicated that tFUS was delivered correctly to the NAc region in the current study.

### 3.3 tFUS elevates functional connectivity between NAc and mPFC

**FIGURE 3:**
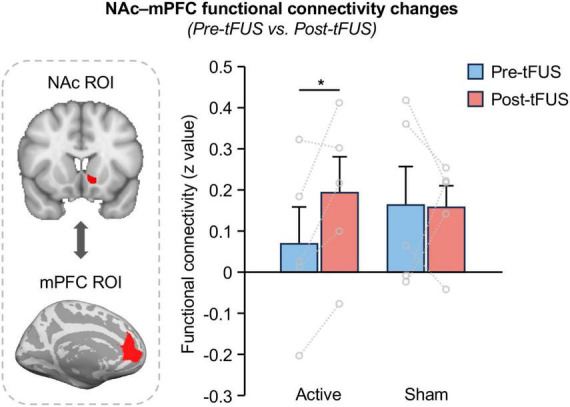
Functional connectivity between NAc and mPFC increases after tFUS on left NAc. A significantly increased functional connectivity between the NAc and mPFC was obtained after stimulating the left NAc in the active group (paired *t*-test, *t* = 2.850, *p* = 0.046), however, no significant changes were observed in the sham group (t = 0.041, *p* = 0.969), indicate that tFUS can modulate NAc functional connections to the mPFC within the reward network. The asterisks (*) indicate significance at *p* < 0.05.

To verify whether tFUS on NAc can modulate the reward circuit, we calculated the FC between the NAc and mPFC, and compared between pre- and post-tFUS in both active and sham groups. We demonstrated a significantly increased functional connectivity between the NAc and mPFC after tFUS on the left NAc in the active group (Figure 3; paired *t*-test, *t* = 2.850, *p* = 0.046), however, no significant changes were observed in the sham group (*t* = 0.041, *p* = 0.969) as well as between the active and sham group at baseline (two-sample *t*-test, *t* = 0.710, *p* = 0.498). This finding indicates that direct tFUS of the NAc can modulate its functional connectivity to other nodes within the reward network. Additionally, we also investigate the FC between NAc and other reward network-related brain regions, including the amygdala, hippocampus, dorsal prefrontal cortex, and insula, however, no significant changes in FC were observed between these regions after tFUS on NAc (Supplementary Figure 2).

## 4 Discussion

To our knowledge, this is the first neuroimage study of delivering tFUS on the NAc in humans to investigate how tFUS modulates brain activity and functional connectivity within the reward network by stimulating the NAc. Our findings revealed that two, 10-min sessions of tFUS delivered to the left NAc administered within the United States Food and Drug Administration (FDA) guidelines of ultrasound power intensity are feasible and safe, with no adverse events related to sonication. Additionally, our findings demonstrated inhibitions of bilateral NAc during tFUS compared to sham. Lastly, we identified increased functional connectivity between the NAc and mPFC after tFUS on the left NAc, but no changes for the sham group.

Nucleus accumbens plays important roles in reward processing, such as driving incentive-based learning, appropriate responses to stimuli, and the development of goal-directed behaviors (Haber and Knutson, 2010). The convergence of afferent, inhibitory, or excitatory, signaling is transmitted to the NAc in a phasic manner and determines its activity (Abler et al., 2006; Carlezon and Thomas, 2009; Sesack and Grace, 2010; Floresco, 2015). The crucial role of the NAc in reward processing makes it a promising target for interventions aimed at modulating dysregulated reward circuitry, such as treatment for SUD. Previous studies on DBS of NAc have demonstrated promising treatment effects in SUD that can greatly reduce substance craving after stimulations (Muller et al., 2013; Coles et al., 2018; Luigjes et al., 2019; Hassan et al., 2021). For this reason, we selected NAc as the target for sonication.

Our findings suggest that tFUS delivered to the left NAc inhibits the brain activation of bilateral NAc compared to the sham group. This result confirmed our hypothesis that delivering tFUS using an inhibitory sonication parameter to the NAc will directly suppress this region. Similar results have also been observed in our previous study by delivering tFUS to the thalamus at the same foundation frequency (Badran et al., 2020). According to early electrophysiological studies, tFUS can have short and long-lasting inhibitory effects on neural pathways, such as suppression of visually evoked potentials (Yoo et al., 2011; Kim et al., 2015) or seizure-like activity in various *in vivo* animal models (Yoo et al., 2010; Min et al., 2011). Furthermore, preliminary studies conducted on healthy human volunteers have revealed a wide spectrum of suppressive and inhibitory effects affecting both cortical and subcortical target regions (Legon et al., 2014; Badran et al., 2020). While the suppression mechanism of tFUS is not yet fully understood, there are several potential explanations, including acoustic cavitation effects, alterations in ion channel permeability, and indirect neuromodulation through membrane deformation (Sanguinetti et al., 2014). However, some other studies (Ai et al., 2018; Gibson et al., 2018) with different parameter settings do show increased activation of the brain. Since tFUS is still a new technique in the field, how tFUS stimulus parameter setting affects whether it is inhibitory or activating needs further investigation.

Additionally, our findings also demonstrated significantly increased functional connectivity between NAc and mPFC after delivering tFUS on the NAc, which is not observed in the sham group. From the non-human primate anatomic tracing study, we know that projections from the mPFC terminate in the NAc (Haber and Knutson, 2010). Although the mechanism underlying the FC increase between NAc and mPFC is still unknown, a possible explanation could be a compensation effect of the inhabitation of NAc by tFUS which leads to a temporally increased communication from mPFC to the NAc. These preliminary findings suggest that tFUS could be potentially a promising neuromodulation tool for the direct and non-invasive management of the NAc and shed new light on the treatment for SUD and other brain diseases that involve reward processing.

There are several limitations in the present study. Firstly, the sample size of the current pilot study is small. Although we used a sham tFUS group as a control for the brain stimulation group, the findings derived from this study could be further verified by larger clinical trials with a more complete demographic distribution. To better address this issue, we performed a power analysis to quantify the main effect of tFUS on mean brain activation within the NAc and the effect size was Cohen’s d = 0.9. Using a similar effect size, a sample size of 21 participants for each group (i.e., active and sham) is sufficient to achieve 80% power with a significance level of *p* < 0.05, which was recommended for future follow-up studies. Secondly, this study was designed as a mechanistic neuroimaging study, and thus we only quantified the imaging measure changes induced by tFUS on the NAc without measuring any behavioral or cognitive scores. Future studies should also investigate the behavioral changes caused by tFUS and their relationship to brain activity and functional connectivity within the reward network. Thirdly, the targeting procedure in this study was carried out by two experienced neuroimagers, and each of them confirmed the targeting location independently to improve the targeting reliability. Additionally, we carried out a control analysis to evaluate the potential shift effect of tFUS on the region/nuclei near the NAc target. Although all the findings supported that tFUS was correctly delivered to the NAc in this study, it is worth noting that there is still a chance that the tFUS targeting could be affected by factors such as the thickness, shape, and structure of the skull. To further improve the targeting accuracy, individual computed tomography (CT) images and spatial navigation systems were recommended to be applied for guiding the target procedure in future studies. Lastly, this study only identified the tFUS-induced brain imaging changes in the healthy population after a single tFUS session. These data suggest that this approach should be explored as a potential intervention for neuropsychiatric disorders that have dysfunctions in reward circuitry, with increased applicability in an SUD population.

## Data availability statement

The original contributions presented in the study are included in the article/Supplementary material, further inquiries can be directed to the corresponding author.

## Ethics statement

The studies involving humans were approved by the Institutional Review Board of the Medical University of South Carolina. The studies were conducted in accordance with the local legislation and institutional requirements. The participants provided their written informed consent to participate in this study.

## Author contributions

XP: Conceptualization, Data curation, Formal analysis, Investigation, Methodology, Project administration, Software, Supervision, Validation, Visualization, Writing – original draft, Writing – review and editing. DC: Data curation, Investigation, Writing – review and editing. FS: Data curation, Investigation, Writing – review and editing. JR: Data curation, Investigation, Writing – review and editing. BB-V: Data curation, Investigation, Writing – review and editing. ES: Data curation, Investigation, Writing – review and editing. BB: Conceptualization, Data curation, Funding acquisition, Investigation, Methodology, Project administration, Resources, Supervision, Validation, Writing – review and editing.

## Footnotes

1
https://surfer.nmr.mgh.harvard.edu/

2
www.fil.ion.ucl.ac.uk/spm/software/spm2/

3
https://fsl.fmrib.ox.ac.uk/fsl/fslwiki
