# Supplementary material for: Non-invasive suppression of the human nucleus accumbens (NAc) with transcranial focused ultrasound (tFUS) modulates the reward network: a pilot study
Source: Front Hum Neurosci. 2024 Apr 2;18:1359396. doi: 10.3389/fnhum.2024.1359396 (PMC11018963; doi:10.3389/fnhum.2024.1359396)
Supplement: Supplementary file 1 [file Data_Sheet_1.PDF]

Supplementary Materials for

Noninvasive Suppression of the Human Nucleus Accumbens (NAc) with Transcranial Focused Ultrasound (tFUS) Modulates the Reward Network: A Pilot Study

Xiaolong Peng, Dillon J. Connolly, Falon Sutton, John Robinson, Brenna Baker-Vogel, Edward B. Short, Bashar W. Badran

<u>This PDF file includes one supplemental figure referenced in the manuscript:</u>

Figure S1

Figure S2

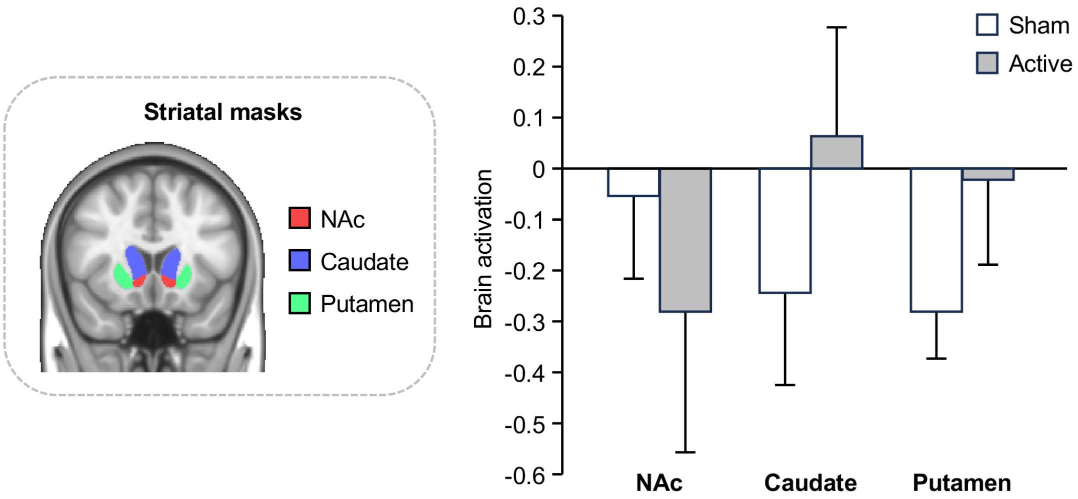

Figure S1. Mean brain activation in striatal masks. To evaluate the accuracy of the tFUS target, we carried out a control analysis to estimate the mean brain activation (contrast "tFUS ON vs. OFF") within three adjacent striatal masks (i.e., NAc, caudate, and putamen) derived from the Harvard-Oxford subcortical template. From the results, brain activations of the NAc decreased in the active tFUS group compared to the sham group, while an opposite trend was observed for the caudate and putamen in which the mean brain activations were higher in the active tFUS group compared to the sham group. Note that, these striatal masks are spatially adjacent to each other, and the tFUS will deactivate the brain region being stimulated due to inhabitation tFUS paraments applied. All the above evidence supported that the tFUS was correctly delivered to the NAc region in this pilot study or at least the main effect of tFUS is concentrated in the NAc region.

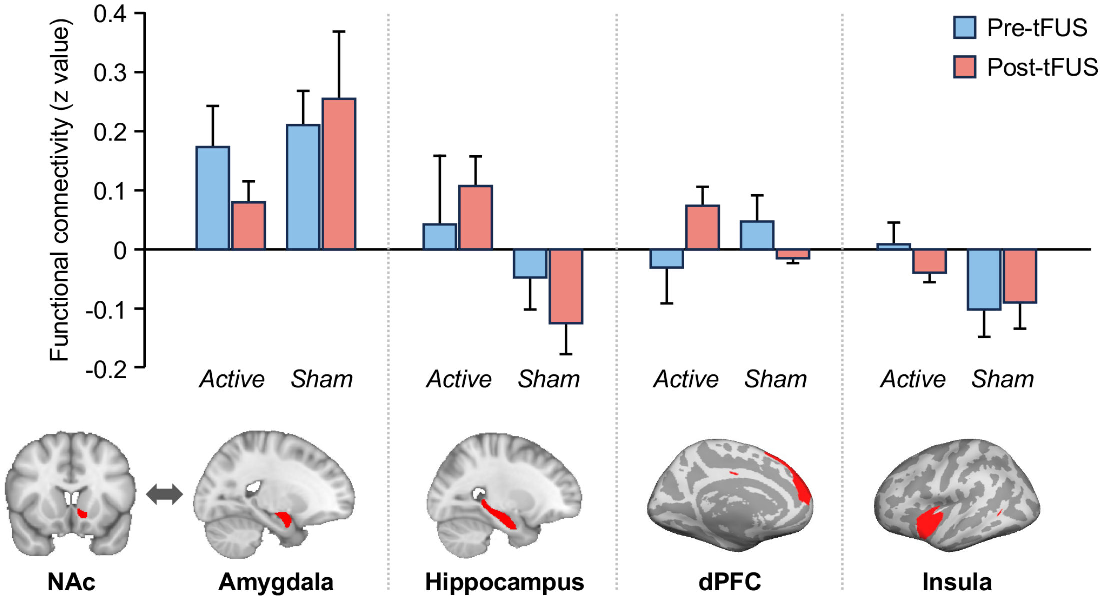

Figure S2. Changes in NAc functional connectivity to other reward network-related regions. We further estimated the functional connectivity between NAc and some other reward network-related brain regions, including the amygdala, hippocampus, dorsal prefrontal cortex (dPFC), and insula. None of these functional connectivities is significantly different between pre- and post-tFUS in both active and sham groups.
